# Diagnostic Dilemma of a Soft Tissue Mass in the Medial Gastrocnemius: A Case Report

**DOI:** 10.7759/cureus.77038

**Published:** 2025-01-06

**Authors:** Fadia Fakhre, Yelena Piazza, Vladimir Neychev

**Affiliations:** 1 General Surgery, University of Central Florida College of Medicine, Orlando, USA; 2 Pathology, University of Central Florida College of Medicine, Orlando, USA; 3 Surgery, University of Central Florida College of Medicine, Orlando, USA

**Keywords:** differential diagnosis, ganglion cyst, gastrocnemius muscle, histopathological examination, intramuscular myxoma, lower extremity mass, mri diagnosis, multidisciplinary approach, soft tissue mass, surgical excision

## Abstract

Soft tissue masses in the lower extremities present significant diagnostic challenges due to the broad spectrum of potential etiologies, ranging from benign to malignant tumors.

A 69-year-old woman presented to the University of Central Florida-Health Surgical Clinic with an enlarging, firm, ovoid mass in her left gastrocnemius muscle causing increasing mostly emotional and psychological distress. A magnetic resonance imaging (MRI) of the left lower extremity was ordered, and findings suggested a 1.8 × 1.8 × 3.1 cm ovoid mass at the proximal medial head of the gastrocnemius muscle with imaging features most consistent with an intramuscular myxoma. However, the differential diagnosis included other benign and malignant entities, such as schwannoma, ganglion cyst, neurofibroma, lipoma, soft tissue sarcoma, Baker's cyst, bursitis, tenosynovitis, abscesses, and vascular lesions.

Surgical excision of the mass revealed a cystic lesion intimately related to the intramuscular portion of the left medial gastrocnemius muscle tendon filled with transparent, gel-like fluid. Histopathological examination confirmed the diagnosis of a ganglion cyst, aligning with the intraoperative findings and providing reassurance of the benign nature of the lesion. The patient's recovery and follow-up were uneventful.

This case underscores the complexities involved in diagnosing soft tissue masses in the lower extremities, particularly when initial imaging findings are not straightforward, suggesting multiple potential etiologies.

## Introduction

Soft tissue masses in the lower extremities present a significant diagnostic challenge due to the broad spectrum of potential etiologies, ranging from benign to malignant lesions [1,2]. Schwannoma, myxoma, neurofibroma, lipoma, soft tissue sarcoma, and Baker's cyst are among the lesions that are on the differential; however, due to similar clinical and imaging features, the precise diagnosis is not always straightforward. Ganglion cyst, another potential diagnosis, is a synovial fluid-filled lesion most associated with a joint capsule or tendon sheath soft tissue mass found within the hand, wrist, and foot. Rarely, ganglion cysts can appear in unusual locations such as muscle aponeurosis and intramuscular portion of the tendon. In such cases, it can be mistaken for other tumors, and the definitive diagnosis is usually established after surgical removal on final pathology [3].

## Case presentation

A 69-year-old female patient presented to the University of Central Florida-Health Surgical Clinic with a firm, ovoid mass located on her left lower extremity at the proximal medial head of the gastrocnemius muscle. She first found the mass a few months prior during the routine application of skin cream to her leg and was uncertain if it had increased in size since its initial discovery. She denied associated pain, changes in skin color, or erythema.

Her past medical history was significant for osteoporosis, vitamin D deficiency, and hyperlipidemia. Her past surgical history included an appendectomy at age 25 and knee arthroscopy for meniscal repair at age 60. There was no family history of similar conditions or malignancies. Socially, the patient is a retired schoolteacher and a non-smoker and does not consume alcohol. She leads a moderately active lifestyle, regularly engaging in walking and yoga.

Physical examination revealed a firm, ovoid mass located in the proximal aspect of the medial head of the gastrocnemius muscle, measuring approximately 3 × 2 cm. The mass was non-tender and relatively immobile and showed no signs of erythema or temperature change compared to the surrounding skin. Peripheral pulses were 2+ for both the posterior tibial and dorsalis pedis arteries. The patient had a full range of motion in the left lower extremity, identical to the unaffected side, with no observed deficits in strength or function. Neurological examination of the limb was normal, with intact sensation and no evidence of paresthesia or muscle weakness. There were no signs of trauma, skin discoloration, or ulceration. There were no palpable lymph nodes in the inguinal region. The remainder of the physical examination, including cardiovascular, respiratory, abdominal, and neurological systems, was unremarkable.

These findings suggested a benign nature of the mass, but given its persistence and the patient's concern, further diagnostic workup was warranted to outline etiology and appropriate management.

**Figure 1:**
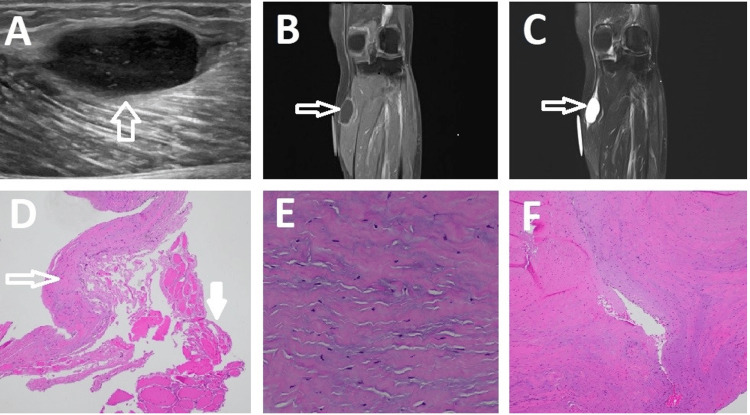
Imaging and Histopathological Features of a Ganglion Cyst in the Medial Gastrocnemius Muscle (A) Sagittal ultrasound image of a well-circumscribed lesion in the left anterior medial calf of uncertain etiology (arrow). (B) Representative coronal MRI image of the mass showing a hypointense signal on T1-weighted sequences (arrow). (C) Representative coronal MRI image of the mass showing fusiform increased signal at the proximal and distal ends of the mass and a thin rim of peripheral enhancement on T2-weighted sequences (arrow). (D) Representative low-power (×40) H&E staining of the lesion, revealing a thin-walled cyst (hollow arrow) with adjacent skeletal muscle fibers (solid arrow). (E) Representative high-power (×200) H&E staining of the lesion with focal myxoid changes of the fibroconnective tissue. (F) Representative low-power (×100) H&E staining of the lesion with slit-like space formation with no true endothelial, epithelial, or synovial lining. MRI: magnetic resonance imaging, H&E: hematoxylin and eosin

The patient had a previous ultrasound (US) of the lower extremity that showed an ovoid hypoechoic lesion in the left medial calf of uncertain etiology (Figure 1A). Due to the uncertainty of the US findings, magnetic resonance imaging (MRI) was suggested as the next imaging modality. The MRI revealed a well-circumscribed mass in the proximal medial head of the left gastrocnemius muscle (Figure 1B, 1C). The initial imaging report described a 2 × 3 cm hyperintense signal on T2-weighted sequences, a hypointense signal on T1-weighted sequences, a fusiform increased T2 signal at the proximal and distal ends of the mass, and a thin rim of peripheral enhancement. The interpretation by the radiologist was that the imaging features were most consistent with an intramuscular myxoma. However, after further discussion with the radiologist, the diagnosis of a schwannoma or another intramuscular tumor could not be excluded. Given the diagnostic ambiguity, a multidisciplinary approach was adopted, involving consultations with radiologists, surgeons, and pathologists. The team agreed that surgical excision would not only provide therapeutic benefit but also allow for a definitive histopathological diagnosis.

The patient underwent an uneventful surgical procedure for the excision of the soft tissue mass from the left lower extremity. Intraoperative findings revealed a well-circumscribed, firm, and relatively immobile mass with limited side-to-side mobility consistent with the preoperative findings. Surgical excision was carried out, taking into account the underlying anatomy adjacent to the cyst to avoid injuring neurovascular structures within proximity to the cystic mass. Upon careful dissection, the mass was noted to be inseparably connected on both apices to the aponeurosis of the medial head of the left gastrocnemius muscle with splayed fascicle-like structures. Because possible schwannoma was high on the differential, the capsule of the lesion was gently and very meticulously incised longitudinally between the splayed fascicle-like structures, and a gel-like fluid was retrieved. This intraoperative finding significantly altered the initial suspicion of a schwannoma, and the filamentous longitudinally running structures initially thought to be nerve fascicles were most likely representing splayed fascicles of the muscle fascia/aponeurosis. The mass was meticulously dissected from the surrounding tissues and excised in its entirety.

Immediate postoperative assessment of left lower limb function and sensation showed no abnormalities.

The final histopathological examination confirmed the diagnosis of a ganglion cyst. The final histopathological examination revealed a thin-walled cystic structure with attached skeletal muscular fibers. The cystic wall demonstrated microscopic myxoid changes within the dense collagenous stroma, while the slit-like cystic lumen showed an absence of epithelial or synovial lining, distinguishing it from a true ganglion cyst (Figure 1D-1F). No malignant cells were identified, reinforcing the benign nature of the lesion. This could be a result of wear and tear processes, trauma, or degenerative changes.

The patient's recovery was uneventful, with no complications observed during the follow-up visits three months after surgery. The surgical site healed well, and there was no recurrence of the mass or any new symptoms.

## Discussion

Diagnosing soft tissue masses in the lower extremities is fraught with challenges due to the wide variety of potential etiologies and overlapping imaging characteristics [3]. In this case, the initial MRI findings suggested a myxoma or possible schwannoma, tumors. However, the differential diagnosis for such a presentation is broad, encompassing benign entities such as ganglion cysts, myxomas, neurofibromas, and lipomas, as well as malignant conditions such as soft tissue sarcomas and vascular lesions [1,2]. In the context of soft tissue masses in the lower extremity, MRI is the imaging modality of choice. It provides detailed information about the lesion's characteristics and its relationship with surrounding structures. Schwannomas typically show well-defined capsules and homogeneous signal intensity, but ganglion cysts, which are fluid-filled, can appear similar on MRI, complicating the diagnosis [4]. Furthermore, atypical presentations of ganglion cysts, such as intraneural or intramuscular locations, can mimic the appearance of schwannomas and other soft tissue tumors, adding to the diagnostic dilemma [5].

This case describes the diagnostic dilemma of a 69-year-old woman with a suspected intramuscular schwannoma of the tibial nerve branch, which was ultimately identified as a ganglion cyst after surgical excision. The accurate diagnosis of soft tissue masses is crucial for appropriate management and treatment, highlighting the need for a multidisciplinary approach involving radiologists, surgeons, and pathologists. Misdiagnosis can lead to inadequate treatment and potentially severe complications. For instance, surgical removal of a schwannoma can result in nerve injury, causing loss of sensation or motor function [4]. Conversely, failing to identify a malignant tumor such as soft tissue sarcoma can delay necessary treatment, leading to disease progression and poorer outcomes [3]. The similarities in imaging characteristics between different soft tissue masses, such as the homogeneity seen in both schwannomas and ganglion cysts, make it easy to confuse these entities [2]. Therefore, a comprehensive diagnostic approach, including detailed imaging and histopathological confirmation, is essential to avoid these pitfalls and ensure optimal patient care.

Ganglion cysts, although typically associated with joint capsules and tendon sheaths, can occasionally present in atypical locations such as intramuscularly or intraneurally, mimicking the imaging features of other soft tissue tumors [6]. These cysts are fluid-filled sacs that can be mistaken for solid masses on MRI, particularly when located deep within the muscle tissue. The definitive diagnosis of ganglion cysts often requires surgical exploration and histopathological confirmation, as their characteristic gel-like fluid content is typically identified intraoperatively [1,5]. In this case, the diagnostic process involved a comprehensive clinical evaluation, detailed imaging studies, and a multidisciplinary approach. The MRI findings of a well-circumscribed, hypoechoic mass with benign features necessitated further consideration of a broad differential diagnosis. The clinical decision-making process emphasized the importance of surgical excision not only for therapeutic purposes but also to achieve a definitive diagnosis through histopathological examination [2].

The intraoperative discovery of the gel-like fluid within the mass made the diagnosis of a cystic lesion (e.g., a ganglion cyst) very likely, underscoring the diagnostic challenges and the necessity of considering multiple potential etiologies when evaluating soft tissue masses. This case highlights the critical role of a multidisciplinary team in the accurate diagnosis and management of such lesions, involving radiologists, surgeons, and pathologists to ensure optimal patient outcomes. The histopathological examination corroborated the benign nature of the ganglion cyst, aligning with the intraoperative findings and providing reassurance to the patient. The successful surgical excision and the patient's uneventful recovery further emphasize the importance of a thorough diagnostic approach and the value of histopathological confirmation in guiding treatment decisions.

Misdiagnosing specific soft tissue masses in the lower extremities can have distinct implications depending on the pathology involved. For instance, failing to accurately identify a soft tissue sarcoma, a malignant tumor, as a benign lesion such as a ganglion cyst or a lipoma can delay critical treatment and allow the cancer to metastasize, significantly reducing survival rates [6]. Conversely, mistaking a benign mass such as a ganglion cyst for a malignant tumor could lead to unnecessary aggressive treatments, including extensive surgical excision or radiation, which come with high morbidity and potential complications [7]. Neurofibromas and schwannomas, both benign peripheral nerve sheath tumors, pose a risk of nerve injury during excision if misdiagnosed and inappropriately managed, potentially leading to permanent sensory or motor deficits [7]. Misidentifying a Baker's cyst or bursitis, which are typically inflammatory conditions, as a neoplastic process could result in unwarranted surgical intervention instead of appropriate conservative treatments such as aspiration or corticosteroid injections [3].

Additionally, misdiagnosing myxomas, which are benign but locally aggressive tumors, as less concerning entities such as lipomas can lead to insufficient surgical margins during excision. This increases the risk of recurrence and potential complications related to incomplete removal [5]. Similarly, identifying a vascular lesion such as a hemangioma or a vascular malformation incorrectly can lead to inappropriate treatment strategies, which might exacerbate the condition or lead to significant bleeding complications [2]. Inaccurate diagnosis of abscesses, which are infectious in nature, as tumors could delay appropriate antibiotic therapy and drainage, resulting in worsening infection and systemic complications [6]. Properly distinguishing tenosynovitis, an inflammatory condition of the tendon sheaths, from neoplastic processes is essential to avoid unnecessary surgeries and instead implement effective anti-inflammatory treatments [7].

Therefore, the accurate categorization of these masses through detailed imaging and histopathological examination is critical in ensuring appropriate management, reducing the risk of complications, and improving patient outcomes [2,8].

## Conclusions

This case underscores the complexities involved in diagnosing soft tissue masses in the lower extremities, particularly when initial imaging findings suggest multiple potential etiologies. The diagnostic ambiguity between schwannomas and ganglion cysts highlights the necessity of a comprehensive diagnostic workup, including detailed imaging and histopathological examination. The multidisciplinary approach involving radiologists, surgeons, and pathologists is important for accurate diagnosis and effective management.

Key clinical teaching points from this case include the importance of considering a broad differential diagnosis for soft tissue masses, the role of MRI in providing detailed characterization of such lesions, and the necessity of histopathological confirmation for definitive diagnosis. This case also illustrates the importance of patient counseling and the need for thorough preoperative planning to ensure successful surgical outcomes.
